# Xmn1-158 γGVariant in B-Thalassemia Intermediate Patients in South-East of Iran

**Published:** 2017-04-01

**Authors:** Ebrahim Miri-Moghaddam, Sara Bahrami, Majid Naderi, Ali Bazi, Morteza Karimipoor

**Affiliations:** 1 Associate Professor, Genetics of Non-Communicable Disease Research Center, zahedan University of Medical Sciences, zahedan, Iran; 2 CardioVascular Diseases Research Center, Birjand University of Medical Sciences, Birjand, Iran; 3 Msc in Biology, Department of Biology, Payame Noor University, Zahedan, Iran; 4 Assistant Professor, Genetics of Non-Communicable Diseases Research Center, Department of Pediatric Hematology & Oncology, Faculty of Medicine, Zahedan University of Medical Sciences, Zahedan, Iran; 5 Msc in Hematology, Faculty of Allied Medical Sciences, Zabol University of Medical Sciences, Zabol, Iran; 6 Ph.D, Molecular Medicine Department, Biotechnology Research Center, Pasteur Institute of Iran, Tehran, Iran

**Keywords:** Xmn-1 polymorphism, β-thalassemia intermediate, Hemoglobin F

## Abstract

**Background:** Xmn-1 polymorphism of 𝜸^G^globin gene (HBG2) is a prominent quantitative trait loci (QTL) in β-thalassemia intermediate (β-TI). In current study, we evaluated the frequency of Xmn-1 polymorphism and its association with β-globin gene (HBB) alleles and Hb F level in β-TI patients in Sistan and Balouchestan province, south-east of Iran.

**Subjects and Methods:** 45 β-TI patients were enrolled. HBB gene mutations and Xmn-1 polymorphism were determined by amplification-refractory mutation system (ARMS) PCR method. Hemoglobin profile was determined using capillary electrophoresis.

**Results:** The study participants consisted of 26 (58%) males and 19 (42%) females. Mean age of the patients was 10.7±3.1 years old. Overall, Xmn-1 polymorphism was observed in 28 (62%) patients. Homozygous (TT) and heterozygous (CT) genotypes of the polymorphism represented with frequencies of 12 (26%) and 16 (35%), respectively. Main recognized HBB gene mutation was IVSI-5(G>C) with homozygous frequency of 44%. Non-zero (β^+^) alleles of HBB gene constituted 11.1 % (4 patients with heterozygous β^+ ^and one with homozygous β^+ ^genotype). Hb F level was significantly higher in patients with at least one Xmn-1allele (67.9±±17.9%) than those without the polymorphism (19.5±20.3%, P<0.0001). Also, patients with homozygous genotype demonstrated significantly higher Hb F compared to heterozygous (CT) cases (respective percentages of 85±±6.8 and 54.7±±10.5, p<0.0001).

**Conclusion: **Our results highlighted the role of Xmn-1 polymorphism as the main phenotypic modifier in β-TI patients in Sistan and Balouchestan province.

## Introduction

 β-Thalassemia intermediate (β-TI) represents a highly heterogeneous entity lying between two extreme forms of β-thalassemia syndromes; β-thalassemia minor and β-thalassemia major.^1^^,^^2^Clinical picture of β-TI ranges from non-symptomatic to severe transfusion-dependent forms. Wide-spectrum phenotypic appearance of β-TI can be partly attributed to its great genetic diversity.^3^^,^^4^ Accordingly, multiple genetic loci are present inside and outside of theβ-globin gene (HBB) cluster which can modulate the clinical severity of β-TI.^5^ However, the main pathophysiological factor determining the severity of β-TI is the ratio of α-globin/non-α-globin chains within erythroid precursors.^5^^,^^6^The majority of the known phenotype modifiers of β-TI execute through counterbalancing the above-mentioned ratio. Multiple genetic polymorphisms within HBB like genes, specific erythroid transcription factors and genes involved in oxido-reductase reactions have been introduced as quantitative trait loci (QTLs) modulating β-thalassemia clinical appearance.^5^ Although the mechanisms exploited by these genetic modifiers are largely obscure, induction of Hb F is considered as an established contributor. 

Xmn-1 polymorphism results from a C > T base substitution at the-158 position of 𝜸^G^ globin (HBG2) gene, and is a well-known HbF inducer ameliorating β-TI severity.^7^This polymorphism resides in close proximity to locus control region of β-globin gene (β-LCR) which controls differential expression of β-like globin genes throughout the life.^8^ Actually, the “T” allele of Xmn-1 polymorphism leads to weaker binding of transcription inhibitors to the β-LCR, and subsequently results in persistent activation of HBG2gene beyond the infancy period.^8^^,^^9^ Studies indicated substantial impact of Xmn-1 polymorphism on improvement of β-thalassemia clinical severity.^10^^-^^12^ Also, there are reports suggesting a role for Xmn-1 polymorphism in predicting the response rateto the Hb F inducer therapeutics in β-thalassemia major. ^8^^, ^^13^^, ^^14^ 

Nevertheless, Xmn-1 polymorphism has demonstrated a variable penetrance among different populations.^15^^,^^16^ In Iranian β-TI patients, this polymorphism has been characterized as the main genetic contributor to the compromised phenotype in β-thalassemia patients.^17^^,^^18^Despite this, there has been no study on the frequency of this polymorphism in Sistan and Balouchestan province in south-east of Iran. Considering that the province is one of the primordial locations of β-thalassemia in the country (with estimated frequency of 2500 registered β-thalassemia major cases),^19^^-^^21^we aimed to evaluate the frequency and clinical significance of Xmn-1 polymorphism in β-TI patients in this region.

## MATERIALS AND METHODS

 The patients (45 cases represented with β-TI) were selected from Ali-Asghar Children Hospital, Zahedan, Sistan and Balouchestan province. These patients have been seeking medical care since their diagnosis in this center. Inclusion criteria were mild symptoms of anemia, intermittent transfusion requirements, and age of starting transfusion >2 years old. Our study was approved by the Research Deputy of Azad University, as well as the Medical Ethics Committee of the Pasteure Institute of Iran. Furthermore, an informed consent was acquired from the patients or their parents. Routine hematological indices were measured by Sysmex K1000 (Japan) blood auto analyzer. Capillary electrophoresis was performed for quantification of HbA_2_ and Hb F.

DNA extraction was carried out using proteinase K method with a standard protocol previously described.^22^Amplification-refractory mutation system (ARMS)-PCR (dNTP cat. No. DN7604C (CinnaGen Company, Karaj-Iran), TaqDNA polymerase Cat. No. TA8109C (CinnaGenCompany, Karaj-Iran)) was conducted to determine the Xmn-1 polymorphism and common HBB gene mutations as previously reported in East of Iran.^20^^, ^^23^^, ^^24^ Furthermore, mutations identified in β-TI patients were further confirmed in patient’s parents. The sequences of the used primers (Biolegio Company, Nijmegen-Netherland) have been presented in Table 1. 

**Table 1:** Sequences of the primers used for detection of Xmn-1 polymorphism

Primer	Nucleotide sequence	Product size (bp)
Forward primer (wild type)	5’-CCAACCCATGGGTGGAGTTTAGCCAAGA-3’	492
Forward primer (mutant)	5’-CCAACCCATGGGTGGAGTTTAGCCAAGG-3’	
Reveres primer (common)	5’-CACTGAAACTGTTGCTTTATAGGATTTT-3’	

## Results

 The current study included 45 unrelated patients diagnosed with β-TI. Mean age of the patients was 10.7±3.1 years old. Detailed demographic and clinical features have been represented in Table 2. The most identified β-globin gene mutations were IVSI-5 (G>C), IVSI-II (G>A) and -88(C>T) with frequencies of 60%, 10% and 6.5%, respectively. Homozygous state for IVSI-5 (G>C) (44%) was the most frequent genotypic combination. Other common genotypes comprised IVSI-5 (G>C)/ -88(C>T) with 8.9%, IVSI-5 (G>C)/IVSII-1 (G>A), homozygous IVSI-II (G>A) and IVSI-5 (G>C)/HbS,each with 6.7% prevalence (Table 3).

**Table 2:** Demographic and clinical features in 45 unrelated β-TI patients

**Parameters**		**Male (n=26)**		**Female (n=19)**	
		**N**	**%**	**N**	**%**
Hb (g/dl)	<7	7	26.9	3	15.7
	7-10	16	61.5	16	84.2
	>10	3	11.5	0	0
MCV (fL)	<80	25	96.1	16	84.2
	>80	1	3.8	3	15.7
MCH (Pg)	<27	23	88.4	18	94.7
	>27	3	11.5	1	5.2
RBC(10^12^/L)	<4.2	16	61.5	12	63.1
	>4.2	10	38.4	7	36.8
Hb A_2_ (%)	<2.5	7	26.9	4	21
	2.5-3.5	5	19.2	5	26.3
	>3.5	14	53.8	10	52.6
Hb F (%)	<5	0	0	5	26.3
	5-50	9	34.6	6	31.5
	>50	17	65.3	8	42.1
Splenectomy	Yes	1	3.8	0	0
	No	25	96.1	19	100
Age of Diagnosis (years)*	Less than 5	13	65	12	70
	Above 5	7	35	5	30

* Data was not available for 8 patients.

**Table 3:** Mutations of β -globin gene were identified in 45 unrelated β-TI patients.

N	Genotype	Type	N	%	Xmn-1 polymorphism			Mean Hb F % (SD)
					CC	CT	TT	
1	IVSI-5/IVSI-5	β^0^/β^0^	20	44.4	12	4	4	45.4(35.9)
2	IVSI-5/IVSII-1	β^0^/β^0^	3	6.7	0`	2	1	60.7(33.9)
3	IVSI-5/-88(C>T)	β^0^/β^+^	4	8.9	1	3	0	47.5(19.3)
4	IVSI-5/Normal	β^0^/β^N^	2	4.4	0	2	0	62.5(49.4)
5	IVSI-5/HbS	β^0^/β^S^	3	6.7	1	2	0	40.1(33.1)
6	IVSI-5/FSC 8/9	β^0^/β^0^	1	2.2	0	1	0	
7	IVSI-5/Del619	β^0^/β^0^	1	2.2	1	0	0	
8	IVSII-1/IVSII-1	β^0^/β^0^	3	6.7	0	0	3	91(6.6)
9	IVSI(-25Del)/Normal	β^0^/β^N^	2	4.4	0	1	1	57.2(30.7)
10	FSC8/9-FSC8/9	β^0^/β^0^	2	4.4	0	1	1	74.3(23)
11	Codo15/Codo15	β^0^/β^0^	1	2.2	1	0	0	
12	-88(C>T)/-88(C>T)	β^+^/β^+^	1	2.2	0	0	1	
13	Unk//Unk	-	2	4.4	1	0	1	41.6(30.1)
	Total		45	100.0	17	16	12	

**Figure 1:**
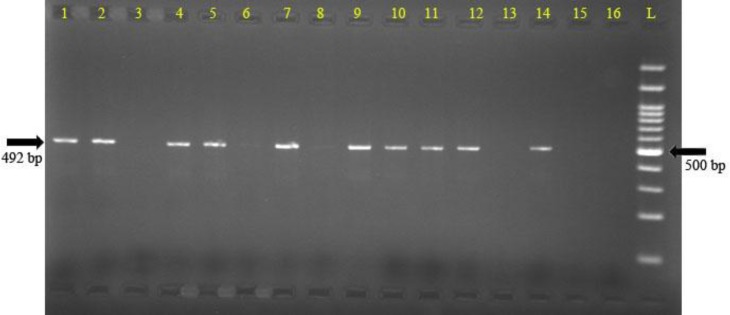
Genotyping of Xmn-1 polymorphism in patients with thalassemia intermediate. The picture demonstrates the results for 7 patients (two well for each patient, the first well for C and the second for T allele). Lanes (1, 2), (3, 4), (5, 6) are controls genotyped as CT, TT and CC respectively. Case 4 (lanes 7, 8) shows the TT genotype, cases 5and 6 (lanes 9 to 12) represent the CT genotype and case 7 (lanes 13, 14) reveals the CC genotype. Lanes 15 and 16 are negative controls. The 100 base pair (bp) ladder line has been depicted by “L”.

Xmn-1 polymorphism was observed in 28/45 (62.2%) of our patients (Figure 1). In 12 (26%) out of 45, a homozygous genotype (TT) was observed, while 16/45 (35%) had heterozygous (CT) status. Mean Hb F was significantly higher (67.9±17.9 %) in the β-TI patients who had at least one Xmn-1 polymorphism in comparison with the patients who represented without the polymorphism (19.5±29.3, P=0.0001). Furthermore, cases with homozygous genotype of Xmn-1 polymorphism had significantly higher mean Hb F percentage (85.5%) than the heterozygous cases (54.7%, P=0.0001, Table 4). 

**Table 4:** Association of Hb F and total Hb values with Xmn-1 polymorphism in 45 unrelated β-TI patients

**Molecular ** **determinant**		**N (%)** **Total=45**	**Hb F (%)**	**Hb ** **(g/dl)**	**Age ** **diag.(years)**
Xmn-1	CC	17 (37.8)	19.5±29.3	7.8±1.5	5.8±3.1
polymorphism	CT	16(35.6)	54.7±10.5*	8.3±1	6.2±5.3
	TT	12(26.7)	85.5±6.8*	8.6±1.6	5.2±5.7

* ; P=0.0001

## Discussion

 Xmn-1 polymorphism is a prominent mediator ameliorating β-thalassemia phenotype through inducing fetal hemoglobin expression.^25^^,^^26^

This polymorphism exhibited 62.3 % frequency in the present study with 35.6% heterozygous and 26.7% homozygous genotypes. In a recent study on 51 Iranian β-TI patients, 68.6% of whom showed CT genotype of Xmn-1 polymorphism, while TT genotype was identified in none of the cases.^8^ In other studies in Iran, Arab et al.^   17 ^ and Akbarietal.^18^ reported the respective Xmn-1 frequencies of 76.9% and 60% in β-TI patients. In the study of Karimi et al. in our neighbor province, Fars, Xmn-1 variant was detected in 40.6% of 48 β-TI patients and 14% of 50 healthy subjects.^   15 ^ In another study in the western province, Kermanshah, 16.3% and 22.3% of patients with severe form of β-thalassemia demonstrated homozygous and heterozygous genotypes of Xmn-1 variant.^27^In studies conducted in Iraq^28^ and Kuwait,^   29 ^ Xmn-1 polymorphism was described in 47% and 75% of β-TI patients, respectively. We observed that Hb F level was significantly higher in patients who had at least one Xmn-1 variant allele than the patients without this polymorphism (67% vs. 19%). This is in consistent with results obtained by Motovali et al. and Galanello et al. ^8^^,^^30^  In addition, we found that the patients who were homozygous for Xmn-1 polymorphism had significantly higher mean Hb F (85.5%) compared to heterozygous subjects (54.7%), which is consistent with the findings from prior works. ^8^^,^^31^^, ^^32^ 

In parallel, Nemati et al. also reported a higher level of HbF in β-thalassemia patients with homozygous genotype of Xmn-1 polymorphism than the ones without this genetic combination.^27^

From molecular perspective, “T” base substitution at Xmn-1polymorphic site is supposed to interfere with interaction of specific transcription inhibitors with regulatory sequences at β-LCR.^8^ This may be suggestive for possible effects of Xmn-1polymorphism in bypassing the attachment of the specific transcriptional inhibitors to the regulatory sequences of HBG2 gene. This idea is further supported by studies indicating that polymorphisms in two main suppressive mediators of HBG2 expression, BCL11A and MYB are associated with moderate clinical picture in β-thalassemia major.^33^^-^^36^ These findings conclusively indicate that main QTLs of β-thalassemia phenotype, including Xmn-1 polymorphism potentially interfere with binding of inhibitory transcription factors responsible for silencing of Hb F expression. This is particularly important for consideration of targeted therapies interfering with interaction of these transcription inhibitors with β-LCR.

Collocation of Xmn-1 polymorphism with specific β-thalassemia alleles have been suggested in β-TI patients. In this regard, a significant association has been described between homozygous state of IVS-II-I (G>A) mutation and Xmn-1 polymorphism by Karimi et al*.*^   15 ^ In line with this finding, we also detected the presence of Xmn-1 polymorphism in all six patients who had at least one IVS-II-I (G>A) allele (Table 2). Along with this, from 20 patients with homozygous IVSI-5(G>C) genotype, 8 (40%) had at least one Xmn-1 allele which may be in part indicative of a relationship between this allele and co inheritance of Xmn-1 polymorphism. Furthermore, Xmn-1 polymorphism was observed in both patients homozygous for FSC8/9 (+G) allele. Nevertheless, the number of our patients with either IVS-II-I (G>A), IVSI-5(G>C) or FSC8/9(+G) mutation was not adequate for exploiting a certain association. Larger population-based studies are recommended to confirm a potential link between certain HBB alleles and Xmn-1 polymorphism. 

It has been suggested that Xmn-1 polymorphism may be restricted to specific β-TI genotypic combinations. Reportedly, the main genetic signature harboring Xmn-1 polymorphism in β-TI patients has been inherited β^0^ alleles.^12^In parallel, we identified the Xmn-1 polymorphism in 17/31 (54%) and 3/4 (75%) of our patient who had β^0^/β^0^ and β^0^/β^+^ signatures, respectively. In accordance with our results, Xmn-1 polymorphism has also been associated with β^0^ thalassemia mutations in 55%-60% of intermediate patients in earlier reports from Iran. ^1^^,^^17^  Likewise, association of Xmn-1 polymorphism with β^0^ mutations reached as high as 80 % in an Iraqi study.^   28 ^ This association was also proposed in the study of Adekile et al., in which Xmn-1 polymorphism coinherited with β^0^ alleles was more frequent than β^+^ alleles in β-TI patients.^29^To sum up, the proposed relationship between inheritance of β^0^ alleles and Xmn-1 polymorphism highlights the role of this polymorphism as a strong modifying factor in severeβ^0^-thalassemia cases. 

There are some reports that are not in accordance with the defined role of Xmn-1 polymorphism in lessening the clinical presentation or boosting Hb F level in β-thalassemia patients. ^31^^,^^37^^-^^39^ 

This notion can be understood from the identification of some patients harboring Xmn-1 polymorphism, and phenotypic picture of β-thalassemia major. ^37^^,^^40^ 

These observations may highlight the impact of some unidentified genetic determinants acting upstream ofXmn-1 polymorphism. On the other hand, neitherXmn-1 polymorphism nor mild β-globin mutations were detected in 13 (27%) of our patients, indicating the possible contribution of other QTLs such as polymorphisms in BCLA11 and HBS1L-MYB transcription factors. ^7^^,^^30^^,^^41^ Morestudies on the molecular aspects of β-TI patients can provide us with a wider view on genetic contributors to the phenotype of β-thalassemia syndromes. Besides, there may be also a possible role for participation of other unrecognized factors acting independent of Hb F induction to alleviate the β-thalassemia phenotype. Since therapeutic strategies aiming to induction of Hb F have largely yielded inconsistent results in β-thalassemia syndromes, identification of Hb F independent mechanisms provides a new promising field of research in this area.

## CONCLUSION

 Our results revealed the Xmn-1 polymorphism as the most prominent molecular basis of β-TI in Sistan and Balouchestan province. However, further studies are recommended for elucidating the possible role of other known QTLs to the better understanding of β-TI molecular basis in our region.
